# Integrated EpCAM-independent subtraction enrichment and iFISH strategies to detect and classify disseminated and circulating tumors cells

**DOI:** 10.1186/s40169-015-0081-2

**Published:** 2015-12-30

**Authors:** Peter Ping Lin

**Affiliations:** Cytelligen, San Diego, CA 92121 USA

**Keywords:** CTC and DTC subtype, Heteroploid chromosome, Tumor biomarker, Metastasis, Liquid biopsy

## Abstract

Application of tumor cell surface adhesion molecule EpCAM-dependent antibody capture, and intracellular cytokeratins (CKs)-dependent immunostaining strategies to detect disseminated or circulating tumor cells (DTCs or CTCs), is limited by highly heterogeneous and dynamic expression or absence of EpCAM and/or CKs in CTCs and DTCs, particularly in their capturing and identifying CTCs/DTCs shed from diverse types of solid tumor, thus being biased and restricted to the only both EpCAM and CK positive cancer cells. Moreover, heterogeneity of chromosome and tumor biomarker of CTCs/DTCs cannot be co-examined by conventional CK/EpCAM-dependent techniques. Accordingly, a novel integrated cellular and molecular approach of EpCAM-independent subtraction enrichment (SE) and immunostaining-FISH (iFISH^®^) has recently been successfully developed. SE-iFISH^®^ is able to effectively enrich, comprehensively identify and characterize both large and small size non-hematopoietic heteroploid CTCs, DTCs and circulating tumor microemboli in various biofluid specimens of either cancer patients or patient-derived-xenograft mice. Obtained tumor cells, free of anti-EpCAM perturbing and hypotonic damage, are eligible for primary tumor cell culture as well as a series of downstream analyses. Highly heterogeneous CTCs and DTCs could be classified into subtypes by in situ phenotyping protein expression of various tumor biomarkers and karyotyping of chromosome aneuploidy performed by iFISH^®^. Each CTC subtype may correlate with distinct clinical significance in terms of tumor metastasis, relapse, therapeutic drug sensitivity or resistance, respectively.

## Background

**Fig. 1:**
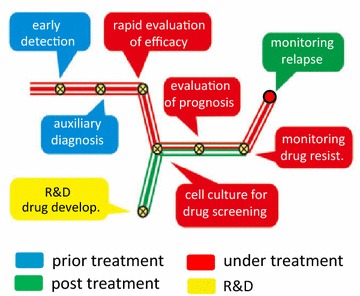
Summary of clinical significance of detecting CTCs. Clinical utilities of detection of CTCs, which are classified into four main categories of prior (*blue*), under (*red*) and post (*green*) therapy as well as R&D (*yellow*), are summarized in the subway map. Distinct significance of CTC is shown at different station

Circulating tumor cells (CTCs) are cancer cells shed from primary or metastatic solid tumors into peripheral blood [1], whereas disseminated tumor cells (DTCs) are neoplastic cells disseminated into biofluid, including bone marrow, ascites, pleural effusion, cerebrospinal fluid (CSF), and urine, etc. [2]. CTCs play a fundamental role in tumor distant metastasis. Clinical utilities of detection of CTCs are summarized in Fig. 1. In particular, quantitative and qualitative examination of CTCs have been applied to rapidly evaluate efficacy of chemo- and targeted therapy, predict prognosis, monitor therapeutic drug resistance and cancer relapse in real time. Detection of CTCs and DTCs is the most representative of “liquid biopsy” due to its unique availability of frequent and non-invasive detecting and monitoring tumor cells in biofluid and peripheral blood of cancer patients. The American Society of Clinical Oncology has accepted quantification of CTCs as a novel breast cancer biomarker [3].

Various methodologies regarding detection of CTCs and DTCs were substantially discussed [4]. An effective detection of CTCs/DTCs is constituted by approaches including both efficient isolation and adequate identification. However, efforts reported to date with respect to enhancing CTCs/DTCs detection have mainly focused on improving either isolation or identification, rarely on both.

### Isolation of CTCs and DTCs

Most of the recognized methodologies for isolating CTCs and DTCs could be classified as cell filtration, antibody capture and enrichment.

#### Cell filtration

The principle of cell filtration (such as ISET) [5] for isolating CTCs relies on the assumption that CTCs are larger than white blood cells (WBCs). Such technique is able to rapidly isolate clusters of CTCs [circulating tumor microemboli (CTM)] and the single CTC only with the size larger than WBCs. However, recent studies demonstrated the existence of plenty of CTCs and DTCs with the size either similar or smaller than that of WBCs in both patients [6–8] or patient derived xenograft (PDX) tumor animal models [7, 9], suggesting that cell size-based filtration may lose significant amount of small CTCs and DTCs [10–12]. Considering CTCs undergoing epithelial-mesenchymal transition (EMT) are smaller in size [13], and many small CTCs are clinically relevant [9, 14], inherent limitation of cell filtration technique on detection of highly heterogeneous populations of CTC and DTC should not be ignored.

#### Antibody capture

Anti-epithelial cell adhesion molecule (EpCAM)-derived technologies, including CellSearch [1] and microfludics or CTC-chip [4, 15], constitute the current antibody capture strategy.

**Fig. 2:**
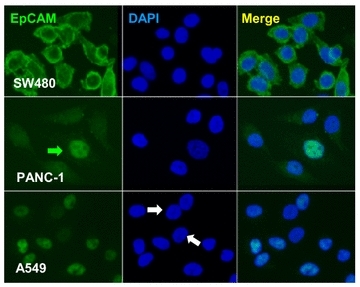
Comparison of EpCAM expression among different types of cancer cell. IF staining of EpCAM was performed on adenocarcinoma cells of colon (SW480), pancreas (PANC-1) and NSCLC (A549). SW480 cells show very high expression of EpCAM on the plasma membrane. Most of PANC-1cells have very low amount of EpCAM localized in cytoplasm and vesicles, and one out of five cells shows EpCAM in nucleus (*green arrow*). All of A549 cells, except two negative in the middle (*white arrows*), have weak nucleus staining of EpCAM. Plasma membrane localization of EpCAM is not visualized on both PANC-1 and A549 cells

Nevertheless, emerging evidence has revealed highly dynamic localization and expression of EpCAM on tumor cells. EpCAM was found on the plasma membrane, in lysosome or nucleus [16, 17]. Intracellular domain of EpCAM could localize in nucleus and plays a fundamental role in signaling pathways [18]. High expression of EpCAM was reported on epithelial neoplastic cells in primary and metastatic lesions, however, low on CTCs derived from solid tumors [16]. Heterogeneous expression of EpCAM on cancer cells among different tissue or even within the same sample was observed [16, 19]. Recent quantitative study performed by flow-cytometry demonstrated that bladder T24 and melanoma SK-Mel-28 cancer cells showed low and non-expression of EpCAM, respectively, compared to that on SK-BR-3 breast cancer cells [7]. Additional extended immunostaining comparison of EpCAM expression illustrated in Fig. 2 showed that only colon cancer cells SW480 had strong EpCAM staining on the plasma membrane, whereas both pancreatic cancer cells PANC-1and non-small cell lung cancer (NSCLC) cells A549 only showed very weak and heterogeneous cytoplasmic and nucleus staining. Though characteristics of cell line cells are not identical to parental tumor cells, revealed insufficient or absence of EpCAM on the plasma membrane may partially account for the ineffective detection of CTCs by anti-EpCAM-dependent strategies in most NSCLC and pancreatic cancer as well as many other types of cancer patients. Interestingly, observed heterogeneous nuclear localization of EpCAM in lung and pancreatic cancer cell line cells in this study seems keeping in accordance with the similar previously published observation on thyroid tumor cells [17].

Besides cancer cell line cells, absence of EpCAM on as high as 30 % of the examined 134 epithelial solid tumors was also reported [20]. Additionally, it has been recently recognized that only EpCAM-negative CTCs in breast cancer patients possess enhanced metastasizing potential to brain [21], though phenotypic existence of EpCAM on metastasis-initiating cells (MICs) among CTC has been demonstrated [22]. Inherit absence or down-regulation of EpCAM which associates with EMT and cancer progression as well as metastasis [21, 23], inevitably result in failure to isolate those “uncapturable” CTCs by means of anti-EpCAM or its derived techniques [19, 24].

Intracellular signaling pathways are triggered following antibody binding and crosslinking of cell surface molecules [25]. It is reasonable to speculate that bio-characterization of CTCs isolated by anti-EpCAM might be perturbed with unpredictable artifacts upon antibody binding to EpCAM, an active signaling molecule on tumor cells [16, 18, 26, 27]. Indeed, anti-EpCAM has been recently reported to induce proliferation and modulate gene expression in human lung cancer cells A549 [28].

#### Enrichment

Negative enrichment is the most recognized enrichment technique for isolating CTCs. The procedure applies both hypotonic lysis to remove red blood cells (RBCs) and anti-CD45 antibody to deplete WBCs at a depletion efficiency range of 2–3 logs [12, 29]. However, significant amounts of post-negative enrichment residual WBCs, deleterious hypotonic damage and loss of CTCs following hemolysis [11, 12, 29], severely interfere subsequent accurate detection, analysis and primary tumor cell culture of CTCs.

Accordingly, a novel enrichment strategy–Subtraction Enrichment (SE), which is distinguished from the conventional negative enrichment, has been developed [7]. Particularly, strategies of non-hemolytic removal of RBCs and application of immunomagnetic beads conjugated to a cocktail of anti-multiple WBC markers antibodies, ensure both minimum hypotonic injury to CTCs/DTCs and maximal removal of WBCs (as high as 4–5 logs). In addition, special coating of the immuno-beads keeps non-specific binding of non-hematopoietic tumor cells to the magnetic particles at minimum. Since SE strategy was reported for the first time to successfully isolate lung cancer CTCs in 2009 [30], substantial improvement has been made to render its maximum efficiency and optimized flexibility for enrichment of CTCs, DTCs and CTM in various specimens of different types of cancer patients [31] or tumor mouse models [9], despite how EpCAM is heterogeneously expressed or cell size is varied.

Rapidly enriched CTCs/DTCs which are unperturbed by antibody and free of hypotonic damage are eligible for primary tumor cell culture (our unpublished results) and several downstream analyses performed on either pooled or single tumor cell.

### Identification of CTCs and DTCs

Currently, nucleic acid analysis and immunostaining of epithelial marker protein [such as cytokeratin (CK)] are the most frequently published techniques for CTC identification.

#### Nucleic acid-based analyses

Nucleic acid-based analyses of tumor biomarkers for detecting CTCs were well summarized [4]. PCR, RT-PCR or next generation sequencing (NGS) have been applied to detect CTC-derived DNA or mRNA in plasma. Recently developed RNA in situ hybridization (RNAish) technique (Affymetrix, Santa Clara, CA, USA) demonstrated its capability for visible identifying mRNA in CTCs. However, availability of the true tumor specific target genes and appropriate interpretation of both positive and negative results still remains a significant challenge and a concern. Moreover, expression and post-translational modification of tumor biomarker proteins, which ultimately play a key biological role in neoplastic cells, cannot be revealed by nucleic acid-based technologies.

#### Immunostaining

Confirmatory immunocytochemistry [7] or immunofluorescent (IF) staining of the intracellular epithelial marker CK currently constitutes the primary CTC identification approach [1]. However, it has been recognized that during EMT, down-regulation of CK is part of an oncogenic pathway that increases tumor invasiveness and metastatic potential [19, 24, 32]. Loss of CKs in tumor cells closely associates with a higher grade and mitotic index in breast cancer patients [32]. Existence of CK negative “invisible” tumor cells significantly interferes precise detection of CTCs and DTCs performed by immunostaining of CKs alone [19, 33, 34]. It is therefore imperative to develop an alternative strategy, regardless of the type and stage of cancer as well as CK expression, to effectively identify heterogeneous CTCs and DTCs.

#### Immunostaining-FISH (iFISH^®^)

Aneuploidy of chromosome(s) in neoplastic cells of different types of cancer has been reported elsewhere. Heteroploid chromosome 8 identified by centromere probe (CEP 8)-FISH was observed in cancer cells from tissue of lung [35], esophageal [36], pancreatic [37], gastric [38], colon [39], bladder [40] and hepatocellular [41] carcinomas, etc. However, the similar FISH approach applied to identify CTCs/DTCs is complicated due to inherent bio-complicacy of hematopoietic WBCs and non-hematopoietic tumor cells [42, 43]. Moreover, similar to nucleic acid-based detection, expression of a series of tumor biomarker proteins on/in CTCs and DTCs cannot be revealed and examined by conventional FISH method.

Currently, EpCAM and CK are taken as epithelial markers for capturing and identifying CTCs/DTCs, respectively [1, 4]. However, heterogeneous expression or absence of EpCAM and CKs on tumor cells restricts relevant technologies to detect those neoplastic cells. Besides being the “epithelial marker”, dual properties of tumor “biomarker” of both EpCAM [16, 18, 26, 27] and CKs [44] have been demonstrated.

Diverse clinical outcomes were found to correlate with quantity of EpCAM expressed on the tumor cells among different types of cancer. In the case of prostate cancer, overexpressed EpCAM associated with progression and distant metastasis [45], whereas increased 10-year survival rate of gastric cancer patients was confirmed to correlate with the increased EpCAM [46]. In contrast, decreased EpCAM was demonstrated to closely correlate with progression, budding and metastasis of both breast and colon cancers [21, 47].

Post-translational modification of intracellular CK18 protein revealed by phenotypic immunostaining, has been reported to correlate with differentiation of hepatocellular carcinoma (HCC) [48]. Down-regulated CK18 protein seemed to promote cell migration [49] and progression of breast [34], nasopharyngeal [44] as well as colon cancers [50], though up-regulated CK18 protein was shown to correlate with poor differentiation, advanced stage, metastasis and recurrence in lung [51], renal cell [52], oral cavity [53], and esophageal squamous cell carcinomas [54]. Similar to the caspase cleaved extracellular CK18 fragment which is a serum biomarker of tumor cell apoptosis [55], its intracellular counterpart, the intact CK18 apparently is an important tumor biomarker with clinical significance. However, characterization of tumor biomarker CK18 and its distinct clinical relevance in CTCs/DTCs have not been specifically addressed previously.

In view of the extraordinary significance in terms of simultaneous phenotyping tumor biomarker protein expression and karyotyping aneuploidy of chromosome(s) in CTCs/DTCs, a novel in situ strategy of immunostaining-FISH (iFISH^®^) combining karyotypic CEP-FISH and phenotypic immunostaining of CD45 as well as tumor markers has been successfully developed to identify non-hematopoietic heteroploid tumor cells [7]. Immunostained proteins in/on CTCs or DTCs are unrestricted to either intracellular or extracellular antigenic epitopes of nuclear, cytosolic or membrane associated tumor biomarkers or epithelial markers [56]. iFISH^®^ technology provides numerous choices for people to target any of the desired tumor biomarkers to be investigated or any of the chromosome to be enumerated or examined.

**Fig. 3:**
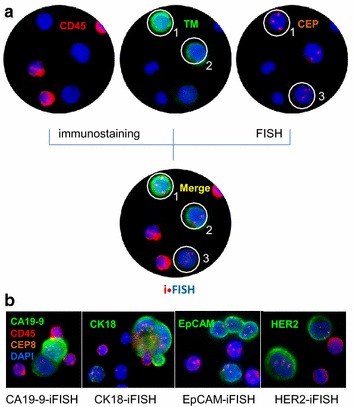
Principle of iFISH. **a** Combined in situ phenotypic immunostaining and karyotypic FISH demonstrate that among 3 CD45 negative non-hematopoietic cells, immunostaining of tumor biomarker (CK18 in this study) alone indicates that *Cell 1* and *2* respectively have high and low CK18 expression, and *Cell 3* has no visible CK18 detected; whereas image of FISH alone performed with CEP of chromosome (chromosome 8 in this study) shows that *Cell 1* and *3* are abnormally triploid, and *Cell 2* are diploid. Merged iFISH image demonstrates that all of *Cell 1–3* are CTCs. *Cell 1* has triploid chromosome 8 with strong CK18 expression; *Cell 2* possesses disomy of chromosome 8 with low CK18 expression; and *Cell 3* shows triploid chromosome 8 with negative CK18 expression. **b** Diverse tumor biomarker-iFISH, including CA19-9, CK18, EpCAM, and HER2-iFISH are illustrated. Experimental protocol of SE-iFISH was previously published [7]. Briefly, 6–8.5 ml peripheral blood, collected into a tube containing acid citrate dextrose anti-coagulant (Becton–Dickinson, Franklin Lakes, NJ, USA), were subjected to centrifuging to remove plasma, followed by centrifuging again on the top of non-hematopoietic cell separation matrix to remove RBCs. Remaining WBCs were incubated with anti-WBC immunomagnetic beads, and subsequently loaded on the separation matrix, then spun down. Cell pellet thoroughly mixed with the cell fixative was applied on the formatted and coated CTC slide. The air dried samples were subjected to FISH probe hybridization and antibody staining performed with Alexa Fluor 594 conjugated monoclonal anti-CD45 and Alexa Fluor 488 conjugated with the indicated antibody [56], followed by image collection and analysis

Principle and diverse types of tumor biomarker-iFISH are described in Fig. 3a. CD45 IF staining was applied to distinguish hematopoietic vs non-hematopoietic cells. Among three of CD45 negative non-hematopoietic cells, additional IF staining showed heterogeneously expressed tumor biomarker (for instance CK18 in this study) in Cell 1 and 2. Whereas FISH examination indicated Cell 1 and 3 had heteroploid chromosome (chromosome 8 in this study). Overlayed iFISH image indicated that instead of two CTCs respectively identified by immunostaining or FISH alone, all of 3 non-hematopoietic cells were CTCs. Trisomy Cell 1 had strong CK18 expression, diploid Cell 2 showed weak expression of CK18, whereas trisomy Cell 3 had no detectable CK18. Obtained results indicate that neither immunostaining nor FISH alone is able to identify all the CTCs which display great phenotypic and karyotypic heterogeneity. Additional different types of tumor biomarker-iFISH, including CA19-9, CK18, EpCAM and HER2-iFISH are revealed in Fig. 3b.

Comparing to current conventional identification approaches, in situ phenotyping and karyotyping of tumor cells performed by iFISH is of particular and unique superiority with respect to detecting various CTCs and DTCs. In addition, iFISH enables classifying CTCs/DTCs into diverse subtypes by in situ phenotyping of the tumor biomarkers and karyotyping of chromosome ploidy (in situ PK CTC or DTC) [7]. A high frequency of CTC subtypes with diverse CK18 expression and aneuploidy of chromosome 8 has been identified and characterized by us in several types of solid tumor including renal cell, HCC, ovarian, colorectal, pancreatic, lung, esophageal and gastric carcinomas [7, 31]. Illustration of the CTCs/DTCs subtypes possessing distinct clinic significance [31] will help guide more specific and significant genotypic, proteomic and functional analyses performed on the targeted single tumor cell [57, 58].

Moreover, in contrast to conventional lengthy FISH protocol which takes more than 20 h, the time required for entire iFISH experiment including antibody staining is as short as 3–4 h, which is very valuable for rapid clinical diagnosis.

### Application of subtraction enrichment (SE)-iFISH

**Fig. 4:**
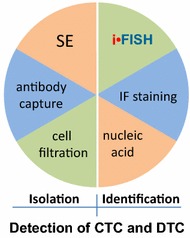
Methodologies for isolation and identification of CTCs or DTCs. Detection of CTCs and DTCs consists of strategies including both isolation and identification. Relative strategies are summarized

Efforts from others to improve CTC detection have mainly focused on either isolation or identification, respectively. However, an effective CTC detection truly relies on both well-established isolation and identification strategies. In view of failure to detect EpCAM negative “uncapturable” and CK negative “invisible” CTCs due to inevitable drawbacks of current EpCAM/CK-dependent methodologies, an integrated tumor cell surface molecule-independent SE-iFISH^®^ platform has been systematically developed and clinically validated (Fig. 4) [7, 9, 31].

Regardless of cellular heterogeneity, inherited down-regulation and/or absence of CKs and EpCAM [4, 59], as well as CTC size variation ranging from similar or smaller than WBCs up to large tumor cells [6, 10, 12], SE-iFISH^®^ enables expeditious detection of CTCs, DTCs and CTMs in regard to efficient enrichment, identification and classification of hypotonic-free, heterogeneous subpopulations of non-hematopoietic heteroploid cancer cells. Our previous and on-going studies showed that those CTCs could be shed from various types of epithelial solid tumor, including lung, glioma, melanoma, osteosarcoma, pheochromocytoma, parathyroid, esophageal, breast, pancreatic, gastric, colon, cervical, ovarian, bladder, renal cell and HCCs in murine or patient’s peripheral blood, or disseminated in bone marrow, CSF, urine, malignant pleural effusion or ascites, despite existence of numerous CK positive mesothelial cells. Obtained viable and native tumor cells free of antibody perturbing are eligible for subsequent primary tumor cell culture (unpublished results) or genetic analyses performed on individual CTC. Successful EGFR mutation analysis performed on the single laser capture micro-dissected (LCM) lung cancer CTC enriched from patients has been recently published [58].

Comparing to conventional EpCAM/CKs-dependent strategy, SE-iFISH^®^ demonstrated higher sensitivity for CTC detection, showing 90.5 % positive rate of SE-iFISH^®^ vs 54.8 % of CellSearch on the identical population of gastric cancer patients [31]. Similar high CTC positivity detected by SE-iFISH^®^ was also observed on lung (92 %) and esophageal (87 %) carcinoma patients [7].

Investigation of how each CTC subpopulation correlates with distinct clinical outcomes is of particular significance. In situ phenotyping and karyotyping analysis of CTC subtypes (in situ PK CTC) performed by iFISH^®^ indicated that among CK18 negative CTCs enriched from gastric cancer patients, trisomy chromosome 8 CTCs may possess intrinsic resistance to the chemotherapeutic agent cisplatin, whereas tetra- and pentasomy subtype developed acquired resistance [31]. Similar results identifying both cisplatin-sensitive and insensitive CTCs in gastric neuroendocrine cancer PDX mice was also recently reported [9]. Of which, CTCs were detected in 200 μl of blood periodically collected for nine times from cisplatin treated or vehicle PDX mice. Cisplatin-sensitive or insensitive CTC subtype could be identified and classified by CK18–iFISH^®^.

Clinical relevance of CTC and DTC subtypes characterized by a number of established tumor biomarkers-iFISH^®^ (such as HER2 [31], CK18, PanCKs, EpCAM, α-fetoprotein [AFP], CD133, CA19.9, Vimentin, etc.) to prognosis, metastasis, drug resistance and recurrence in large cohorts of diverse types of carcinoma patient is currently under our active investigation. Subsequent NGS genetic analyses and comparison of the targeted single CTC or DTC vs neoplastic cells in primary and metastatic lesions will provide very valuable insights for people to understand and illustrate mechanisms of tumor metastasis.

It is anticipated that SE-iFISH^®^ could help promote more specific and significant analyses on pooled or single CTC/DTC, and may also help establish polyclonal or even monoclonal patient CTC/DTC subtype-derived “xenograft”(CDX or DDX) mouse models [60].

## Conclusion

In view of highly heterogeneous and dynamic expression or absence of EpCAM and CK in CTCs and DTCs, the epithelial marker-independent SE-iFISH^®^ platform provides additional choice and flexibility, with higher sensitivity and specificity, to detect various CTCs/DTCs without being restricted and biased to the only both EpCAM and CK positive neoplastic cells. In situ phenotyping of tumor biomarker expression and karyotyping of chromosome ploidy performed by iFISH^®^ will shed light on additional intriguing clinical utilities and significance of diverse subtypes of CTC/DTC, and will also help guide more meaningful studies performed on the targeted single tumor cell enriched from different types of cancer patient or tumor animal models.
