# Erratum to: Integrated EpCAM-independent subtraction enrichment and iFISH strategies to detect and classify disseminated and circulating tumors cells

**DOI:** 10.1186/s40169-016-0085-6

**Published:** 2016-02-09

**Authors:** Peter Ping Lin

**Affiliations:** Cytelligen, San Diego, CA 92121 USA

## Erratum to: Clin Trans Med (2015) 4:38 DOI 10.1186/s40169-015-0081-2

The abstract of the original article in *Clinical and Translational Medicine* [1] contained an error. In the first sentence the words “Anti-epithelial cell adhesion” were incorrectly added. Find the incorrect and corrected sentences below.

*Incorrect sentence:*

Application of tumor cell surface adhesion molecule Anti-epithelial cell adhesion molecule (EpCAM)-dependent antibody capture, and intracellular cytokeratins (CKs)-dependent immunostaining strategies to detect disseminated or circulating tumor cells (DTCs or CTCs), is limited by highly heterogeneous and dynamic expression or absence of EpCAM and/or CKs in CTCs and DTCs, particularly in their capturing and identifying CTCs/DTCs shed from diverse types of solid tumor, thus being biased and restricted to the only both EpCAM and CK positive cancer cells.

*Corrected sentence:*

Application of tumor cell surface adhesion molecule EpCAM-dependent antibody capture, and intracellular cytokeratins (CKs)-dependent immunostaining strategies to detect disseminated or circulating tumor cells (DTCs or CTCs), is limited by highly heterogeneous and dynamic expression or absence of EpCAM and/or CKs in CTCs and DTCs, particularly in their capturing and identifying CTCs/DTCs shed from diverse types of solid tumor, thus being biased and restricted to the only both EpCAM and CK positive cancer cells.

The abstract in the original article has been corrected.
